# The 20k Samples-Per-Second Real Time Detection of Acoustic Vibration Based on Displacement Estimation of One-Dimensional Laser Speckle Images

**DOI:** 10.3390/s21092938

**Published:** 2021-04-22

**Authors:** Nan Wu, Shinichiro Haruyama

**Affiliations:** Graduate School of System Design and Management, Keio University, 4-1-1 Hiyoshi, Kohoku-ku, Yokohama, Kanagawa 223-8526, Japan; haruyama@sdm.keio.ac.jp

**Keywords:** laser speckle image, line-scan sensor, image processing, real time system

## Abstract

Audio signal acquisition using a laser speckle image is an appealing topic since it provides an accurate and non-contact solution for vibration measurement. However, due to the limitation of camera frame rate and image processing speed, previous research could not achieve real time reconstruction of an audio signal. In this manuscript, we use a one-dimensional laser speckle image to measure the acoustic vibration of sound source and propose a fast and sub-pixel accuracy algorithm to estimate the displacement of captured one-dimensional laser speckle images. Compared with previous research, the proposed method is faster and more accurate in displacement estimation. Owing to this, the frequency bandwidth and the robustness are significantly increased. Experiment results show that the proposed system can achieve 20k samples-per-second sampling rate, and the audio signal can be reconstructed with high quality in real time.

## 1. Introduction

Using optical means to measure the physical vibration caused by sound source is an appealing research topic. The optical vibrometers can accurately measure sub-wavelength mechanical vibrations without contacting objects. These characteristics allow the technology to have broad application prospects in various fields, such as optical microphone [1,2,3], surveillance of audio signal in rescue [4,5,6], visual accelerometer [7], and so on.

Generally, optical vibrometers utilize light interference to measure the vibration of objects. The representative instrument is laser Doppler vibrometers (LDVs) [8,9,10]. In the last few decades, with the invention of digital cameras and the improvement of computer performance, there were many studies on vibration measurement which combine optical sensing and image processing technologies [11,12,13]. These studies can be divided into two categories according to the light source utilized. One way is using natural lighting illumination, and the representative research is the visual microphone proposed by researchers from MIT in 2014 [14]. In this research, the authors used a high-speed camera to record the video of a vibrated object and used a phase-based algorithm to extract the audio signal from the captured video. This approach does not require additional illumination, which makes system structure simple and easy to implement. However, processing the video containing the details of the object vibration requires a high computational cost. Slow image processing speed is a disadvantage of this method. Another way is using coherent light illumination. Specifically, when the laser beam illuminates an object of interest, the scattered light interferes in space, forming a speckle pattern. When the object vibrates, the speckle pattern presents a corresponding movement. The camera captures the dynamic speckle pattern rather than the object itself, and the vibration information of the object can be extracted by analyzing the motion of the captured speckle images. In the past, the digital image correlation (DIC) technique was well used for speckle displacement analysis [15,16,17,18,19,20]. However, DIC usually suffers from two disadvantages: (1) up-sampling of the correlation coefficients is required to achieve sub-pixel precision displacement estimation, which is very time consuming; (2) it requires a certain size of speckle pattern to provide a stable accuracy of displacement estimation. The slow computational speed makes it hard to fulfill real time reconstruction of an audio signal using the DIC method. Another technique extracts object vibration using the gray value variation of the speckle image [21,22,23]. This approach uses a special algorithm to filter out the appropriate seed points, obtaining the gray value variation of each point and carrying out data fusion to reconstruct the audio signal. However, this method requires a speckle pattern with a linear intensity distribution in the direction of motion. It is difficult to perform well when the speckle brightness is low or the image size is small.

In our previous research, we used a small image size to improve frame speed of a conventional industrial camera and calculated the optical flow of the captured speckle images [24,25]. The camera captures over 2000 frames per second with the image size of 32 × 32 pixels, and the average time consumption is 1 millisecond for optical flow calculation. Compared with the DIC method, we successfully realized the real time reconstruction of audio signals with the frequency up to 500 Hz. This approach has greatly improved the sampling speed of the laser-speckle measurement system. However, due to the limitations of both conventional area-scan camera’s frame rate and image processing speed, it is still difficult to regenerate high frequency audio signals in real time. For example, the requirement for sampling human speech is at least 8 kHz. The real time sampling rate of previous methods cannot satisfy this requirement [26].

In this manuscript, we propose using a line-scan camera to further improve the sampling speed of the sensing system. The line-scan camera usually can capture tens of thousands of 1D images per second, which greatly improves the detectable frequency range. Besides, using 1D images also brings the reduction of the computational cost of image processing. To estimate the motion of captured 1D images, a fast and accurate displacement estimation algorithm is proposed. The algorithm calculates 1D optical flow and estimates the global displacement of the captured speckle images. The results show that the proposed algorithm can achieve a microseconds level’s calculation speed and an accuracy with the average error less than 0.03 pixels. Audio recovering experiment results show high frequency audio signals, such as human speech, can be detected and restored in real time with high quality by our system.

The structure of this paper is as follows. The explanation of the speckle sensing model along with our displacement estimation algorithm are given in Section 2. Then, the experiment results of our system, including the performance of our proposed displacement estimation algorithm and the results of audio signal detection, are shown in Section 3. Finally, the conclusion of the paper is given in Section 4.

## 2. Materials and Methods

### 2.1. Speckle Sensing Model

**Figure 1:**
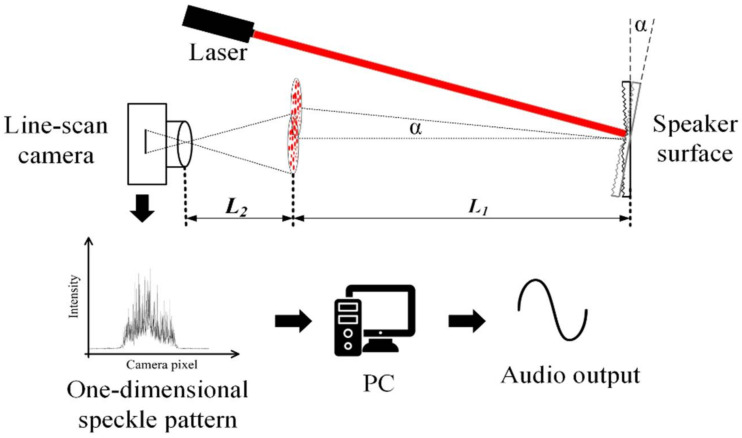
Schematic of the real time audio signal detection system using 1D laser speckle images.

The sketch of our proposed system is shown in Figure 1. An infrared laser beam (λ=980 nm, power=50 mW) illuminates on the surface of a speaker. Although the quantum efficiency of the image sensor is low at the wavelength of 980 nm, the laser light is also invisible to human eyes. The optical microphone system using such a light source does not emit annoying light, which makes it suitable for commercial application. The scattered speckle pattern is captured by a line-scan camera (Photonfocus MV1-L2048-96-G2, Lachen, Switzerland) with a lens (Avenir TV zoom lens, f=75 mm, Tokyo, Japan). In our system, the camera lens is focused on the plane away from the object determined by distance $L_{1}$. The camera captures 1D laser speckle images with a frame rate of 20 kHz. The captured speckle images are processed by a common desktop PC (AMD Ryzen 5 3600 processor, 3.60 GHz, Sunnyvale, America) to output the restored audio signal.

In the system, the vibration of the speaker surface can be regarded as a rigid body motion. Generally, the rigid body motion has three motion modes: in-plane transverse, axial motion, and tilting. Previous research proved that the displacement of the speckle pattern is mainly determined by the tilting of the object if the imaging system is strongly defocused [15]. Assume the tilting angle of the object is α, then the shift amount D of the speckle pattern at the distance $L_{1}$ from the object can be expressed as:

(1) $$D=L_{1}\tan\alpha$$

The magnification of the imaging system is $M=\frac{L_{2}-f}{f}$. Therefore, the image shift d at the image sensor is:

(2) $$d=D\times \frac{1}{M}=\frac{L_{1}f}{L_{2}-f}\tan\alpha$$

Since the tilting angle of the object is usually very small, and $L_{2}$ is much larger than f, the expression of d can be simplified and approximately written as:

(3) $$d=\frac{L_{1}}{L_{2}}f\alpha$$

It can be seen that there is a linear relationship between the image shift and the tilting angle of the object. When the object vibrates, the speckle pattern shows linear vibration at the image sensor. This means it is possible to use a line-scan camera to restore the vibration information of the object, such as the amplitude and the frequency of the vibration, by analyzing the motion of the captured speckle images.

### 2.2. Explanation of 1D Optical Flow Algorithm for Displacement Estimation

In our system, the camera’s sampling rate is 20 kHz under line-scan mode. For such a high frame rate, there are two requirements for the real time motion estimation algorithm. First, the algorithm’s calculation speed must be fast enough to catch up with the camera’s frame rate. Besides, the displacement between two adjacent images is usually less than one pixel. The proposed algorithm must have sub-pixel accuracy.

**Figure 2:**
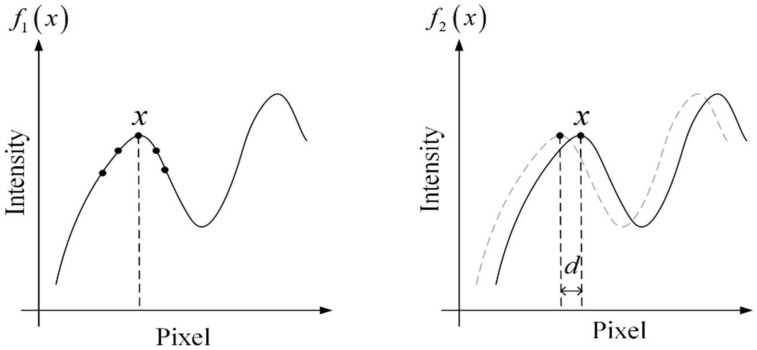
Local signal model of the 1D image undergoes a small shift.

Based on these considerations, a displacement estimation algorithm using 1D optical flow is proposed. Figure 2 shows the signal model of the 1D image, where image intensity is regarded as a quadratic function $f\left(x\right)$ of the pixel location. For example, for pixel x in image $f_{1}\left(x\right)$, the local signal model can be expressed by doing quadratic polynomial fitting using the neighborhood of pixel x, which is:

(4) $$f_{1}\left(x\right)=a_{1}x^{2}+b_{1}x+c_{1}$$

Assuming the displacement is d, by doing polynomial fitting, we have the local signal model $f_{2}\left(x\right)$ at the corresponding pixel in the shifted image, which is:

(5) $$f_{2}\left(x\right)=a_{2}x^{2}+b_{2}x+c_{2}$$

It should be noted that $f_{2}\left(x\right)$ is created by shifting $f_{1}\left(x\right)$ with the amount of d. Therefore, $f_{2}\left(x\right)$ can be also expressed as $f_{1}\left(x-d\right)$. Expanding the expression, we have:

(6) $$\begin{array}{ll} f_{2}\left(x\right) & =f_{1}\left(x-d\right)\\ & =a_{1}{\left(x-d\right)}^{2}+b_{1}\left(x-d\right)+c_{1}\\ & =a_{1}x^{2}+\left(b_{1}-2a_{1}d\right)x+a_{1}d^{2}-b_{1}d+c_{1} \end{array}$$

In this way, the polynomial coefficients of the shifted image are connected to the ones from the original image. Generally, the shift amount is very small between two captured frames because of the high sampling rate of the camera. Assuming the intensity is constant in two images, the coefficients in the two polynomials are equal:

(7) $$a_{1}=a_{2}$$

(8) $$b_{1}-2a_{1}d=b_{2}$$

(9) $$a_{1}d^{2}-b_{1}d+c_{1}=c_{2}$$

By solving Equation (8), we can determine the value of d, which is:

(10) $$d=-\frac{1}{2a}\left(b_{2}-b_{1}\right)$$

where $a=\frac{a_{1}+a_{2}}{2}$.

Repeating this process, the displacement of all pixels can be calculated. In other words, we can obtain a dense optical flow between two 1D images. However, due to the image noise and the deviation of quadratic fitting, pixelwise solutions of Equation (10) do not give good results. The values of displacement at each pixel are very different. On the other hand, a known fact is that, for rigid body, the small object motion does not change the speckle pattern but only shifts the pattern by a small amount [27,28]. Each pixel’s motion on the speckle should be the same. Therefore, in our proposed algorithm, we calculate the global displacement of the speckle images based on the obtained dense optical flow.

Rewriting Equation (10), let $\Delta b=-\frac{1}{2}\left(b_{2}-b_{1}\right)$. At each pixel, we have:

(11) a×d=Δb

Assuming the captured image has N pixels, we can calculate the array of coefficients $a_{1}$, $b_{1}$ from $f_{1}\left(x\right)$ and $a_{2}$, $b_{2}$ from $f_{2}\left(x\right)$ for each pixel. In this way, we can build N equations such as Equation (11). Here, we try to find d satisfying N equations as well as possible. In other words, our goal is minimizing the following function:

(12) $$e=\sum_{i=1}^{N}\left|a_{i}\times d-\Delta b_{i}\right|$$

In conclusion, the actual calculation process in the program is as follows. The two input images are preprocessed to reduce the high-frequency noise. After that, quadratic polynomial fitting is performed on the two images pointwise to obtain the coefficients. Then, the coefficients arrays a and Δb are obtained. Finally, the least square solution of displacement d is determined, which can be expressed as:

(13) $$d=\frac{\sum_{i=1}^{N}a_{i}\Delta b_{i}}{\sum_{i=1}^{N}a_{i}^{2}}$$

## 3. Experiment Results

### 3.1. Performance Test of 1D Optical Flow Algorithm

**Figure 3:**
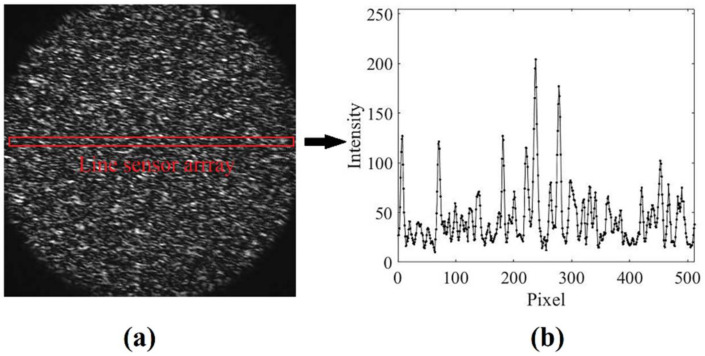
Captured speckle images; the shutter time was 20 μs. (**a**) Captured two-dimensional speckle pattern with the size of 512 × 512 pixels. (**b**) Captured 1D speckle pattern with the size of 1 × 500 pixels.

Before conducting experiments of audio signal detection, the computational accuracy and the speed of the proposed 1D optical flow algorithm are investigated. In this manuscript, the speaker was used as the test object. The material of the speaker surface was resin, and the speaker surface was diffusive to laser beam. Here, we illuminated the infrared laser on the center of the speaker surface. The diameter of the laser spot on the speaker surface was 6 mm. Due to the diffusive surface of the speaker, multiple lights were scattered randomly, and a high-contrast speckle pattern could be observed by camera because of the interferometry of the reflection lights. Figure 3a shows a captured 2D speckle pattern with the size of 512 × 512 pixels, whereas Figure 3b shows the captured 1D speckle pattern by the line-scan sensor. Here, we converted the 2D image to the frequency domain and added a linear phase to manually produce a known subpixel shift along the horizontal direction [29]. Then, an image template with a size of 1 × 500 pixels from the origin image and the shifted image were selected to do the displacement estimation. Since the intensity distribution of speckle pattern was stochastic, the average error on 200 random templates was carried out to improve the accuracy of error estimation.

**Figure 4:**
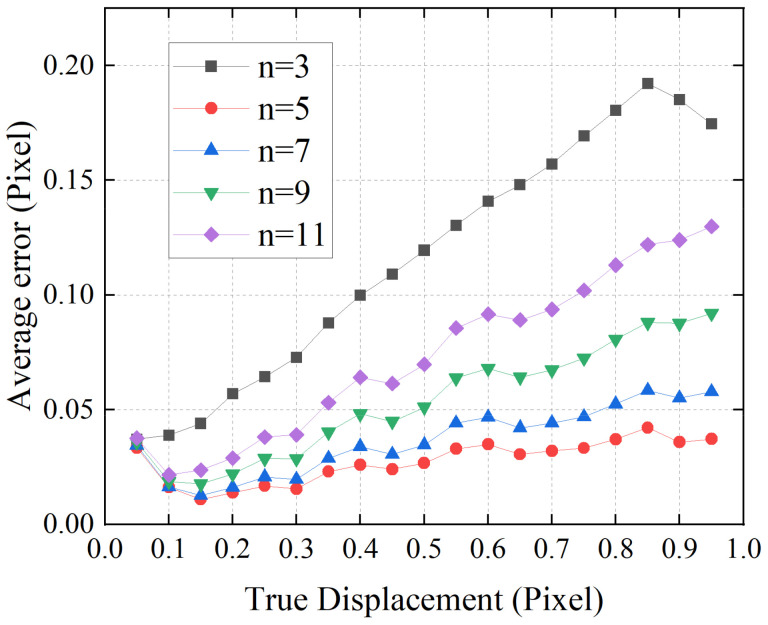
Dependence of the average error result on the shift amount for different quadratic polynomial fitting kernel size.

First, the relationship between the number of pixels n used in quadratic polynomial fitting and the accuracy of displacement estimation was investigated. The proposed algorithm performs quadratic polynomial fitting on the local 1D speckle image, thus the optimal number of pixels used in polynomial fitting is dependent on the average size of the speckle. If the number is too large or too small n reduces the fitting accuracy and results in bad results of displacement estimation. Here, we manually shifted the image from 0.05 pixels to 0.95 pixels with a step of 0.05 pixels. Figure 4 presents the dependence of the average error on shift amount for different polynomial kernel size n. The results showed that polynomial fitting using 5 pixels provided the best accuracy on displacement estimation. If the polynomial kernel size was small (e.g., n=3), the result showed the largest error on displacement estimation. This is because the kernel size was too small to provide accurate polynomial fitting of a local signal. On the other hand, if the kernel size was larger than the speckle size, spatial aliasing of speckles within the polynomial kernel could be an issue, which also affects the accuracy of polynomial fitting. One can see that, when the kernel size was larger than 5 pixels, the accuracy of displacement estimation decreased as the kernel size increased. Speckle size is determined by many factors, such as the wavelength and the diameter of the laser beam, the roughness of the surface, and the defocusing of the imaging system. In our situation, the 5 pixels polynomial fitting was adopted for further tests since it showed the best performance on displacement estimation.

**Figure 5:**
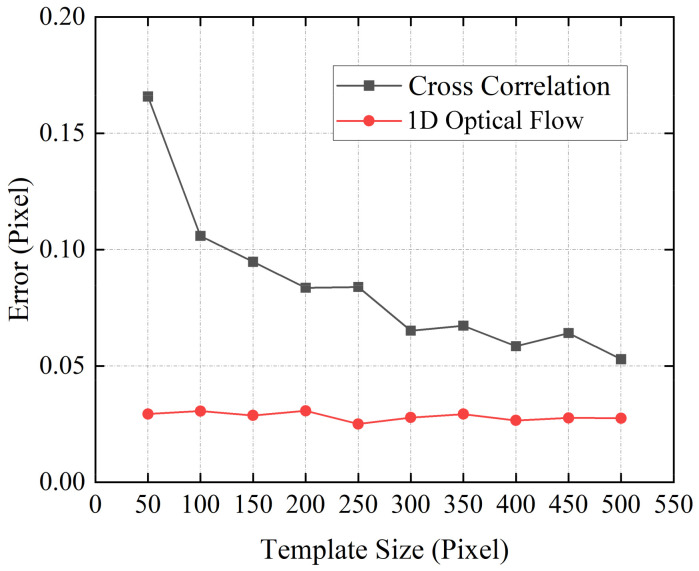
Dependence of the average error of displacement estimation on the template size for different algorithms.

In the next experiment, the displacement estimation accuracy using a different image template size was investigated. As a comparison, the performance of a well-used cross-correlation algorithm was also investigated. This algorithm uses cross-correlation in Fourier space and employs an up-sampled matrix-multiplication DFT to achieve arbitrary subpixel precision [30]. Here, we set the subpixel precision to 100, which meant the resolution of the cross-correlation algorithm was 0.01 pixel. Figure 5 shows dependence of the average error of displacement estimation on the template size for the two algorithms. From the results, we can see that the 1D optical flow algorithm gave better results than the cross-correlation method. Moreover, it should also be noted that the error of cross-correlation algorithm gradually increased as the template size decreased. When the template size was 1 × 50 pixels, the accuracy of displacement estimation was not stable. On the other hand, the proposed 1D optical flow algorithm always gave good calculation accuracy under different template sizes.

**Figure 6:**
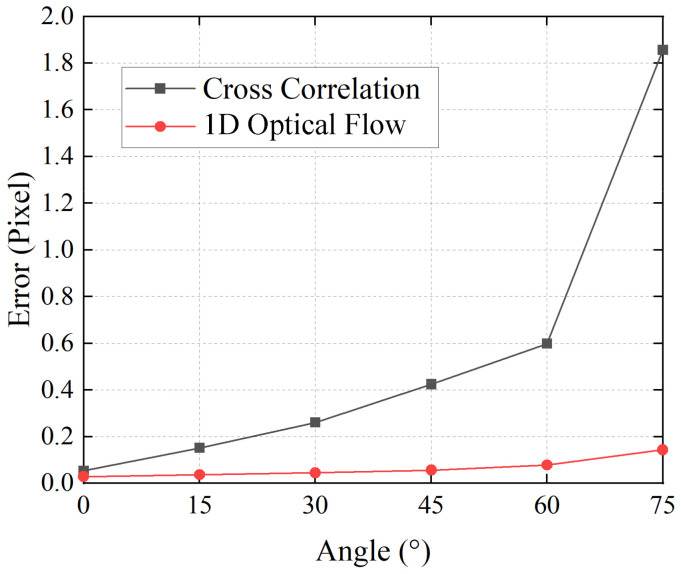
Dependence of the average error of displacement estimation on the angle for different algorithms.

In our system, line sensor is used to observe the global shift of speckle image, and the direction of image vibration is supposed to correspond with the sensor array. However, in actual situations, it is difficult to perfectly match the directions of the line sensor and the speckle motion. The angle between the two directions may result in noise of the displacement estimation. Therefore, in the next experiment, we tested the performance of the two algorithms when the speckle shift was inconsistent with the line sensor array. We used the same method to manually shift the image with a known amount along different directions. Then, we used a set of image templates with the size of 1 × 500 pixels to calculate the shift component in the horizontal direction and obtain the average error. Figure 6 presents the dependence of the average error on the angle for the two algorithms. It can be seen from the result that the inconsistency between the line sensor array and the vibration direction of the speckle image resulted in increasing errors in displacement estimation. Especially for the cross-correlation algorithm, the average error rapidly increased as the angle increased. However, the calculation by the 1D optical flow algorithm showed better robustness than the cross-correlation method. The average error was less than 0.05 pixels when the angle was less than 45°. In an actual situation, the proposed 1D optical flow algorithm is more robust in estimating the horizontal displacement when the image does not vibrate along the line sensor array.

**Figure 7:**
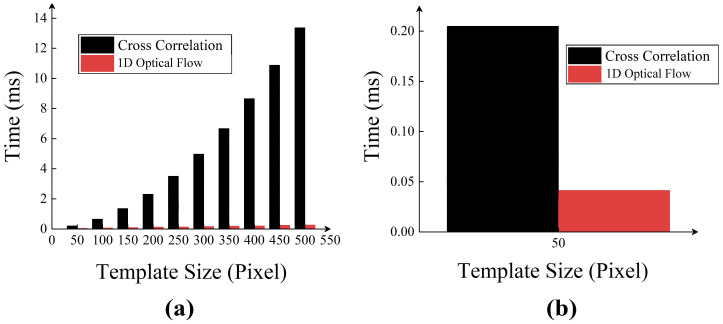
Comparison of average time consumption of two algorithms. (**a**) Dependence of time consumption on template size. (**b**) Time consumption of two algorithms with a template size of 1 × 50 pixels.

Last, we investigated the computational speed of the two algorithms. Here, 20,000 times displacement calculations were conducted for both the 1D optical flow algorithm and the cross-correlation algorithm, and the average time consumption with different template size is shown in Figure 7a. It can be seen from Figure 7a that using smaller size templates reduced the computational load and achieved a higher calculation speed. The test results also showed that the proposed 1D optical flow algorithm was much faster than the cross-correlation algorithm. Especially when the template size was 50 pixels, as shown in Figure 7b, the 1D optical flow algorithm took only 41 μs for displacement estimation, whereas the cross-correlation method required 205 μs. This meant that the calculation speed of the proposed algorithm matched the sampling speed of the camera to achieve a real time acquisition and processing rate of 20 kHz. According to the Nyquist sampling theorem, our system can sample the signal frequency up to 10 kHz in real time. This sampling speed can satisfy the requirement of human speech sampling.

### 3.2. Result of Real Time Audio Signal Extraction

**Figure 8:**
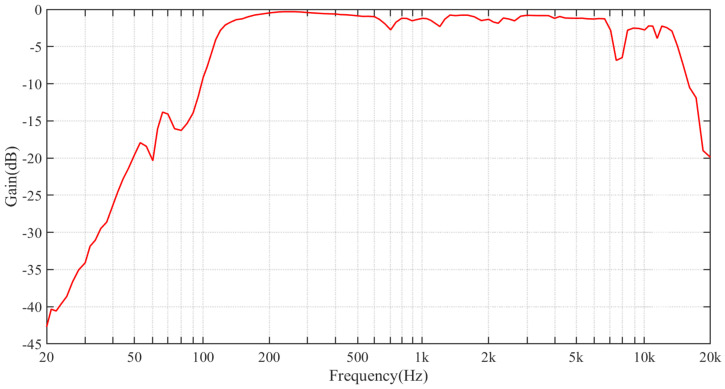
Frequency response of the speaker used in the experiments.

In the next experiment, we tried to use our system to extract a single frequency audio signal of a speaker. Before conducting the experiment, we first investigated the frequency response of the speaker. We made a sound-absorbing box using sound-absorbing materials and put the speaker and the microphone in it to make the result as accurate as possible. The speaker played an audio signal with increasing frequencies from 20 Hz to 20k Hz. The recorded signal was analyzed to obtain the efficiency of the speaker for different frequencies, as shown in Figure 8.

**Figure 9:**
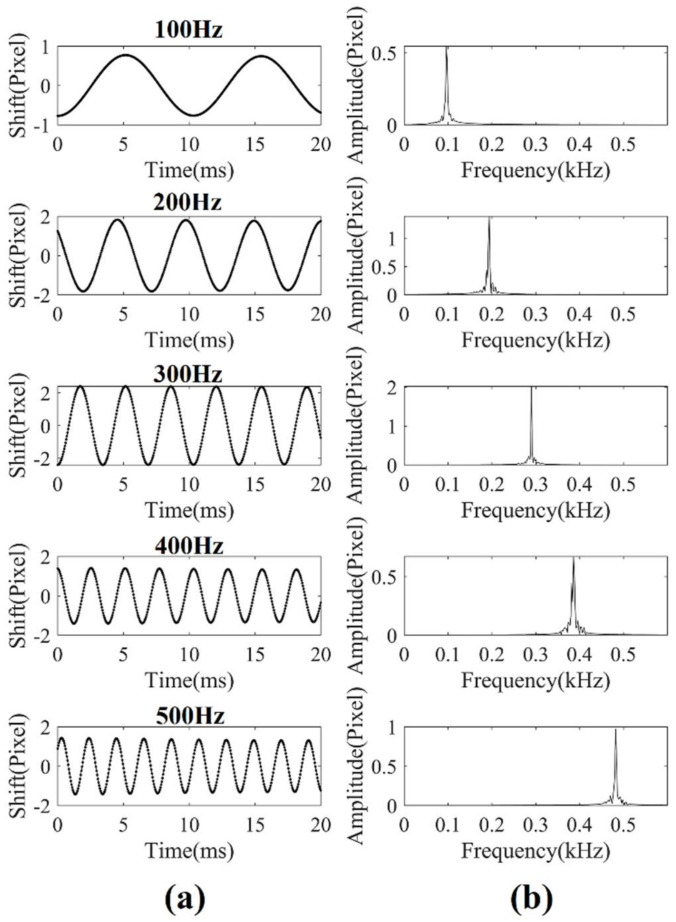
(**a**) Restored waveforms of audio signals at different frequencies. (**b**) Spectrogram of restored signals at different frequencies.

**Figure 10:**
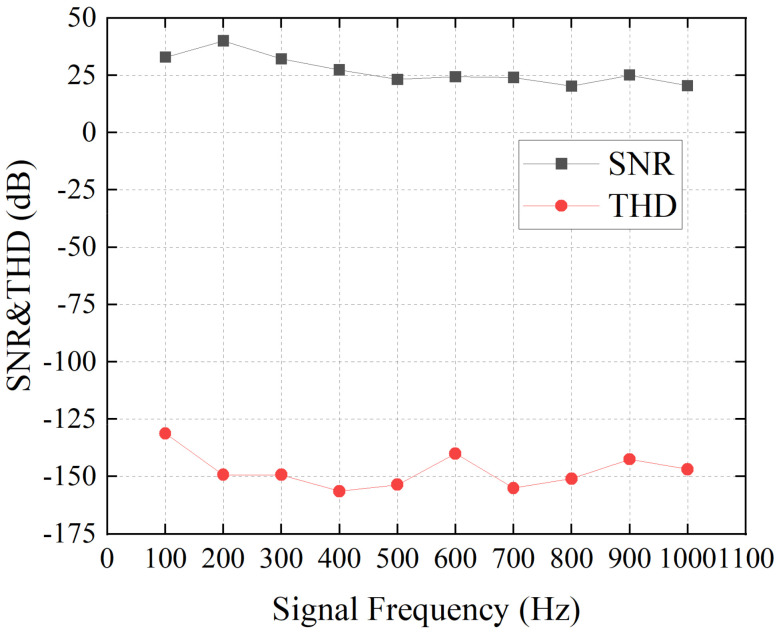
SNR and THD of the restored signal with different signal frequency.

Here, we used a region of 1 × 50 pixels of the line-scan camera. The line-rate was 20,000 frames per second, and the shutter time was 20 μs. The speaker was positioned 2 m away from the camera. The captured images were processed in real time, and the results were exported. First, we sent the signal with constant frequencies in the speaker. Figure 9a presents the first 20 ms waveform of the reconstructed signal with the frequency of 100 Hz to 500 Hz. The waveform was the calculated shift of the observed speckle motion on the line sensor. Figure 9b shows the spectrogram of the results. The results matched the signal that was sent. It should be noted that the amplitude of the 100 Hz regenerated waveform was smaller than other signals, because the frequency response of the speaker was low at 100 Hz frequency. The performance of our system was also investigated using the signal-to-noise ratio (SNR) and the total harmonic distortion (THD). SNR is defined as the ratio of signal power to noise power. A larger SNR means better signal quality. On the other hand, THD is defined as the ratio of the sum of the powers of all harmonic components to the power of the fundamental frequency. A lower THD means a more accurate reproduction of an audio signal. The results obtained with increasing frequencies from 100 Hz to 1000 Hz are shown in Figure 10, which prove that the sinusoidal audio signal could be restored with high quality using our system.

**Figure 11:**
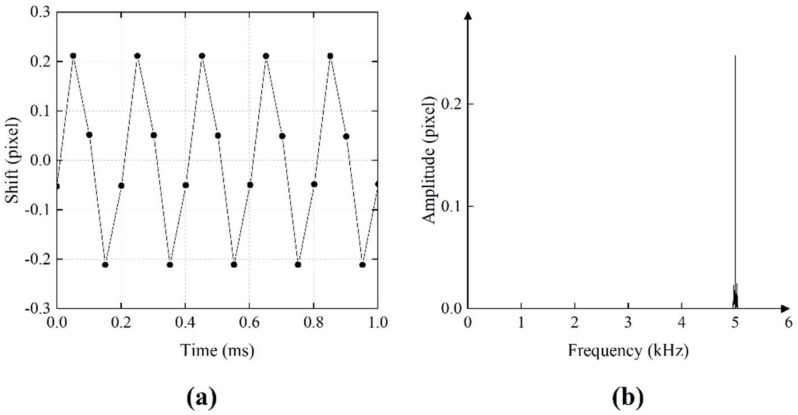
(**a**) The first 1 ms of restored temporal signal; the original signal frequency was 5 kHz. (**b**) Spectrogram of the restored audio signal.

In the next experiment, we tried to extract a signal with high frequency. This time, the 5 kHz sinusoidal signal was played by the loudspeaker. We used the camera to take 10,000 frames and simultaneously analyzed the displacement between captured frames to restore the waveform of the signal. The result of the restored temporal signal is presented in Figure 11a. In Figure 11b, we present the spectrogram of the reconstructed signal. The results prove that the high-frequency vibration of the object could be observed with a speckle image of 1 × 50 pixels, and our algorithm restored the frequency and the amplitude information of the high-frequency vibration correctly in real time. The SNR was 12.64 dB for the result. It should be noted that the restored high frequency signal had a lower signal-to-noise ratio compared with the low-frequency signal. This is because the amplitude was low when the speaker vibrated at high frequency, which resulted in the reduction of the SNR.

**Figure 12:**
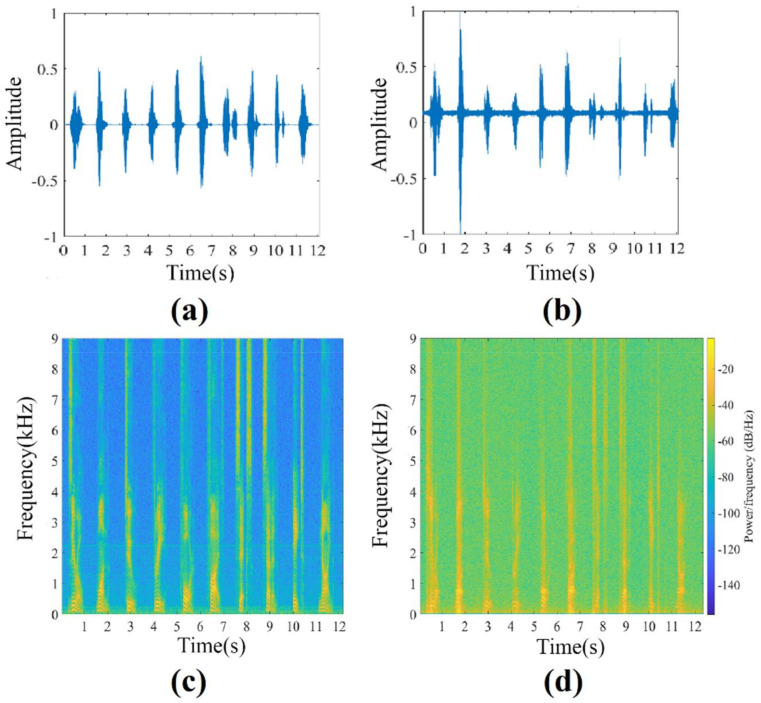
Experiment result of restoring human speech in real time. (**a**,**b**) Temporal signal of the original sound and the restored sound. (**c**,**d**) Spectrogram of the original and the restored sound. (see also Supplementary Materials at the end of the paper).

Finally, we present the result of human speech extraction with our system. Figure 12a shows the temporal signal of the original sound, which was the voice of a male counting from zero to nine in English. Figure 12b shows the temporal signal of the restored sound. Figure 12c,d show the spectrograms of the original and the restored audio signals. The experiment showed that the contents of human voice could be regenerated clearly in real time owing to the high sampling rate of the proposed system. For reference, the audio files of both original music (see Supplementary Materials Audio S1) and regenerated music (see Supplementary Materials Audio S2) are provided as the result of this experiment.

### 3.3. Further Discussion

In Section 2, we provide an explanation of the laser speckle sensing model. In our system, a line-scan camera is adopted to sense the speckle motion. This brings increasing frequency bandwidth. However, one disadvantage of this approach is that a line-scan sensor can only observe speckle motion in one-dimensional format, whereas the speckle vibration is a two-dimensional motion. Usually, the speckle image vibration direction is not perfectly consistent with the line sensor array. Here, the experiment was conducted to investigate the performance of audio signal recovery using our algorithm when the speckle did not move along the line sensor. For comparison, the performance of the cross-correlation algorithm was also investigated.

**Figure 13:**
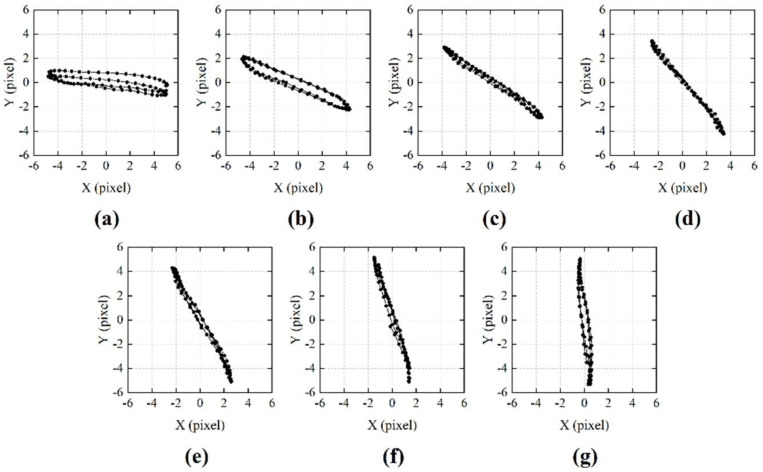
Trajectories of the 2D dynamic speckle patterns with different rotation stage angles. (**a**) Stage angle was 60°. (**b**) Stage angle was 75°. (**c**) Stage angle was 90°. (**d**) Stage angle was 105°. (**e**) Stage angle was 120°. (**f**) Stage angle was 135°. (**g**) Stage angle was 150°.

**Figure 14:**
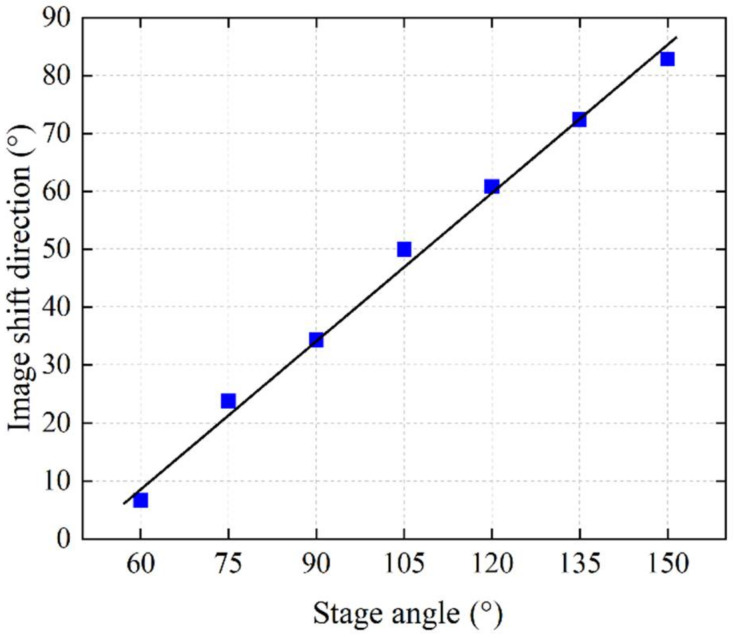
Dependence of the 2D speckle image shift angle and the rotation stage angle.

To obtain dynamic speckle patterns with different vibration directions, the speaker playing an audio signal with a frequency of 100 Hz was fixed on a rotation stage. By changing the angle of the rotation stage, we could control the vibration direction of the captured 2D speckle patterns. Figure 13 shows the trajectories of the captured 2D dynamic speckle patterns with different rotation angles. The image shift directions could be estimated from the trajectories, which were expressed as the absolute value of the angle (acute angle) between the speckle motion direction and the horizontal direction. Table 1 and Figure 14 show the relationship between stage angles and the 2D image shift directions. From the result, we can see that, when the stage angle was 60°, the image shift direction was almost consistent with the line-scan sensor’s direction. As the angle of the rotation stage increased, the image shift direction also changed linearly. When the stage angle was 150°, the 2D image shift direction was almost orthogonal to the line sensor array.

**Figure 15:**
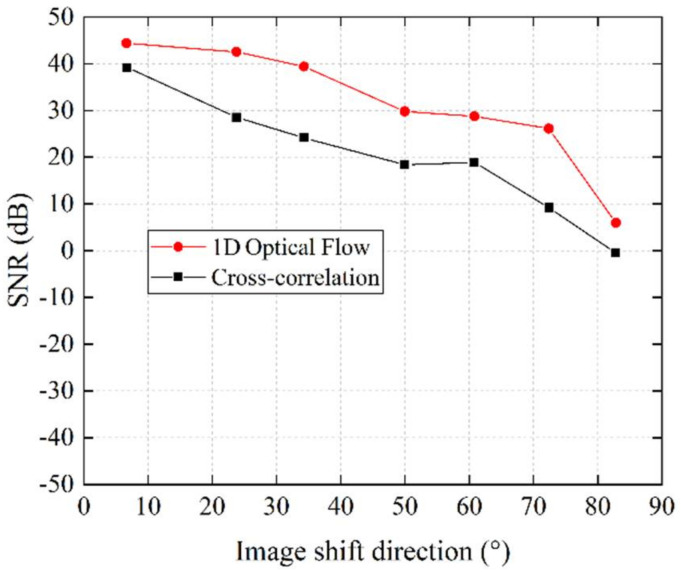
Dependence of the SNR of the restored audio signal on the 2D image shift angle for different algorithms.

**Figure 16:**
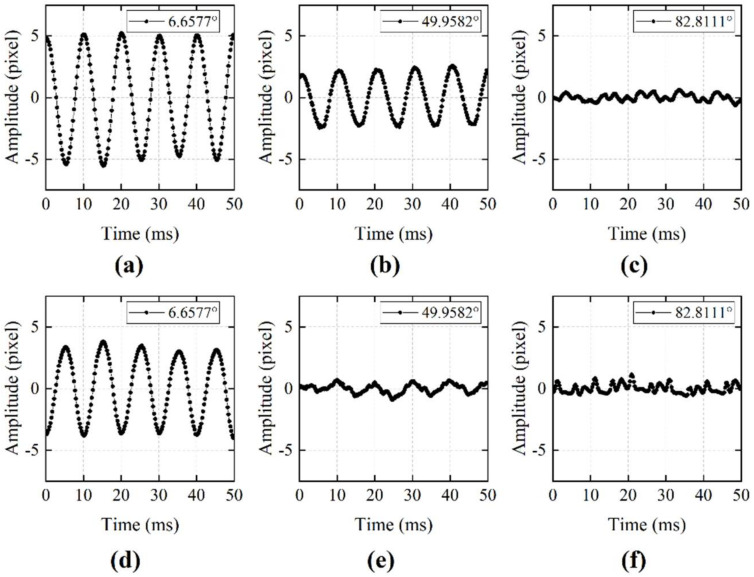
Restored waveform with different 2D image shift angle using different algorithms. (**a**–**c**) Restored waveform using 1D optical flow algorithm. (**d**–**f**) Restored waveform using cross-correlation algorithm.

Next, the camera was switched to line-scan mode to conduct audio signal recovering tests. The captured speckle images were analyzed both with our algorithm and the cross-correlation algorithm to restore the audio signal. Figure 15 shows the SNR results of both algorithms with different situations. Figure 16 shows the waveform of the restored signal. From the results, we can see that, when the angle between the 2D image movement direction and the line sensor was small, both algorithms performed well and restored the audio signal with high quality, as shown in Figure 16a,d. As the angle increased, the cross-correlation algorithm could not give stable results. The SNR dropped rapidly, and the restored waveform was distorted, as shown in Figure 16e. On the other hand, our algorithm showed a better result than the cross-correlation method. As shown in Figure 16b, when the image shift angle was 49.9582°, our algorithm still calculated the shift amount in the horizontal direction correctly and restored the information of the audio signal with high quality. When the image shift direction was orthogonal to the line sensor’s direction, neither algorithm could restore the audio signal, as shown in Figure 16c,f. The experiment results showed that our algorithm had stronger robustness for the disadvantage that the line sensor can only observe the speckle movement in one dimension. Even when the speckle motion direction was not consistent with line sensor array, our algorithm could still restore high-quality audio signals within a certain range by calculating the horizontal motion component.

## 4. Conclusions

In this manuscript, we proposed a fast motion estimation of a 1D laser speckle image and showed its application on a real time audio signal detection system. The major contribution of our work is the fast displacement estimation algorithm for 1D speckle images. Owing to this, the proposed system can achieve a 20k Hz real time sampling speed. Experimental results presented the capabilities of the proposed system for extraction of high frequency audio signals and human voice. The high speed, real time sampling system discussed in the manuscript has broad application prospects, such as voice signal acquisition and high frequency vibration monitoring of industrial equipment.

## Figures and Tables

**Table 1:** Relationship between rotation stage angle and the 2D image shift angle.

**Stage angle (°)**	60	75	90	105	120	135	150
**2D image shift angle (°)**	6.6577	23.7615	34.2741	49.9582	60.7933	72.3964	82.8111

## Data Availability

Not applicable.

## Supplementary Materials

The following are available online at https://www.mdpi.com/article/10.3390/s21092938/s1, Audio S1: Original audio file, Audio S2: Recovered audio file.
